# Cancer-Associated Fibroblasts Promote Migration and Invasion of Non-Small Cell Lung Cancer Cells *via* miR-101-3p Mediated VEGFA Secretion and AKT/eNOS Pathway

**DOI:** 10.3389/fcell.2021.764151

**Published:** 2021-12-16

**Authors:** Xueru Guo, Mengmeng Chen, Limin Cao, Yiming Hu, Xueqin Li, Qicheng Zhang, Yinghui Ren, Xiang Wu, Zhaowei Meng, Ke Xu

**Affiliations:** ^1^ Tianjin Key Laboratory of Lung Cancer Metastasis and Tumor Microenvironment, Tianjin Lung Cancer Institute, Tianjin Medical University General Hospital, Tianjin, China; ^2^ Department of Toxic Laboratory, Tianjin Medical University General Hospital, Tianjin, China; ^3^ Core Facility Center, Tianjin Medical University General Hospital, Tianjin, China; ^4^ Department of Nuclear Medicine, Tianjin Medical University General Hospital, Tianjin, China

**Keywords:** cancer-associated fibroblasts, lung cancer, metastasis, miR-101-3p, VEGFA

## Abstract

Cancer-associated fibroblasts (CAFs) are major component of tumor microenvironment (TME), which plays crucial roles in tumor growth, invasion and metastasis; however, the underling mechanism is not fully elucidated. Despite many studies are focused on the tumor promoting effect of CAFs-derived cytokines, the upstream regulators of cytokine release in CAFs is largely unknown. Here we found that miR-101-3p was downregulated in primary lung cancer-associated CAFs compared to normal fibroblasts (NFs). Ectopic overexpression of miR-101-3p suppressed CAFs activation, and abrogated the promoting effect of CAFs on migration and invasion of non-small cell lung cancer cells (NSCLC), through attenuating CAFs’ effect on epithelial mesenchymal transition (EMT) process, metastasis-related genes (MMP9, TWIST1) and AKT/endothelial nitric oxide synthase (eNOS) signaling pathway. Further study indicated that vascular endothelial growth factor A (VEGFA) was a novel target of miR-101-3p, and CAFs-derived VEGFA mediated the effect of miR-101-3p on migration and invasion of lung cancer cells, demonstrated by using recombinant VEGFA and VEGFA neutralizing antibody. Interestingly, the analysis of the Cancer Genome Atlas (TCGA) database revealed that lung cancer tissues expressed lower level of miR-101-3p than non-cancerous tissues, and low/medium-expression of miR-101-3p was associated with poor overall survival (OS) rate. Moreover, the mouse xenograft experiment also showed that CAFs accelerated tumor growth whereas miR-101-3p diminished CAFs’ effect. These findings revealed a novel mechanism that CAFs facilitated lung cancer metastasis potential *via* miR-101-3p/VEGFA/AKT signaling pathway, suggesting miR-101-3p as a potential candidate for metastasis therapy.

## Introduction

Lung cancer is the main course of cancer-related mortality worldwide. According to the report of the World Health Organization (WHO), there were estimated 2.2 million new lung cancer cases and 1.8 million deaths in 2020 (Sung et al., 2021). The major subtype of lung cancer is NSCLC which accounts for 85% of total lung cancer. Almost 90% of lung cancer patients die of invasiveness and metastasis, results in the 5-year survival rate of lung cancer is only 15% (Cataldo et al., 2011). Therefore, the investigation on the key regulators of metastasis is critical for the improvement of lung cancer treatment.

Emerging evidence reveals that TME plays pivotal roles in tumor initiation and development *via* regulating immune escape, inflammation, angiogenesis and therapy response. The composition of TME is complex, including tumor cells, stromal cells, extracellular matrix (ECM), blood vessels and lymph-vessels (Altorki et al., 2019). CAFs are one of the major stromal cells in TME. CAFs support and promote tumor progression via secreting cytokines and growth factors. CAFs-derived transforming growth factor-β (TGF-β) induced EMT and promoted aggressive phenotypes in breast cancer (Yu et al., 2014). CAFs upregulated CXCR4, β-catenin, PPARδ, and enhanced invasiveness of lung adenocarcinoma by secretion of stromal cell-derived factor (SDF-1) (Wang Y. et al., 2021). Zhang et al. (2019) reported that human colorectal cancer-derived CAFs stimulated adhesion of colorectal cancer cells to endothelial cells via releasing hepatocyte growth factor (HGF). Our previous studies also showed that CAFs facilitate metastasis and chemoresistance of lung cancer cells through IL-6 and ANXA3 secretion (Wang et al., 2017; Wang et al., 2019). Despite many studies are focused on the promoting effect of CAFs-derived cytokines on cancer cells, the upstream regulators of cytokine release in CAFs is largely unknown.

MicroRNAs (miRNAs) are a class of small non-coding RNA with 20–22 nucleotides. MiRNAs bind to 3′-UTR of target mRNAs through complementary base-pairing, and negatively regulate target genes at transcription level via perfect complementarity or at translation level *via* imperfect complementarity. MiRNAs play important roles in various cellular processes including cell proliferation, differentiation, apoptosis and survival (Esquela-Kerscher and Slack, 2006). In particular, miRNAs are also involved in tumor invasion and metastasis. MiR-153-5p promotes the proliferation and metastasis via targeting AGO1 in renal cell carcinoma (Li et al., 2021), our previous study demonstrated that miR-26a enhances invasiveness of human lung cancer cells by suppressing GSK3β (Lin et al., 2017). On the contrary, miRNAs may also inhibit metastasis. MiRNA-32-5p inhibits EMT and metastasis in lung adenocarcinoma by targeting SMAD3 (Zhang J.-X. et al., 2021), miR-16-1-3p suppresses breast cancer growth and metastasis by inhibiting Warburg Effect (Ye et al., 2020).

Given the roles of CAFs in tumor progression and metastasis, we in the present study, explored the role of miRNA in CAFs’ promoting effect. We found that miR-101-3p was downregulated in lung cancer-associated CAFs. We further demonstrated that downregulation of miR-101-3p in CAFs increased VEGFA secretion, facilitating the metastasis potential of lung cancer cells via activation of Akt/eNOS signaling pathway.

## Materials and Methods

### Reagents and Antibodies

The miR-101-3p mimics, inhibitor and control were obtained from Genepharma (Shanghai, China). Human recombinant VEGFA and VEGFA neutralizing antibody were purchased from R&D Systems (Minneapolis, MN). The antibodies against VEGFA, Vimentin, AKT, p-AKT, eNOS, p-eNOS, MMP-9, TWIST1, N-cadherin were purchased from Cell Signaling Technology (Beverly, MA). The antibody against β-actin was purchased from Sigma-Aldrich (St Louis, MO). The antibody against E-cadherin was purchased from BD Bioscience (San Jose, CA). The antibody against α-smooth muscle actin (α-SMA) was purchased from Abcam (Cambridge, United Kingdom).

### Lung Cancer Cell Culture

Human lung cancer cell lines A549, H1299 and H661 were obtained from American Type Culture Collection (Manassas, VA). A549 cells were cultured in DMEM medium (GIBCO BRL, Grand Island, NY). H661 and H1299 cells were grown in RPMI1640 medium (GIBCO). Medium were supplemented with 10% fetal bovine serum (GIBCO). All cells were maintained at 37°C under 5% CO_2_.

### Isolation and Culture of Lung Stromal Fibroblasts

Lung cancer-associated fibroblasts (CAFs) and normal lung fibroblasts (NFs) were isolated and cultured, and conditioned medium (CM) were collected after 48 h according to the method previously described (Wang et al., 2017). The tumor tissues and adjacent normal tissues were obtained from the NSCLC patients underwent surgery at Tianjin Medical University General Hospital (TMUGH; Tianjin, China). The informed consents were obtained from patients. The study was approved by the Institutional Review Board of TMUGH.

### Cell Proliferation

Lung cancer cells were plated in a 96-wells plate at a density of 5 × 10^3^ cells/well. The cells were cultured with same amount of conditioned medium for 48 h and the cell viability was determined by the CCK-8 kit (Dojindo, Kumamoto, Japan).

### Cell Migration

Wound healing assays were performed to examine the ability of cell migration as previously described (Li X. et al., 2020). Cells were plated in 6-well plates and cultured with same amount of different CM. A plastic pipette tip was used to make a clean wound area across the well when cells grew to 100% confluence. Cells were allowed to migrate in the medium. Pictures were taken by a microscope (Nikon, Tokyo, Japan) after 24 h to estimate the wound closure.

### Cell Invasion

Transwell assay can be a method to examine the cell invasion ability as previously described (Cao et al., 2020). Chambers (Corning, Tewksbury, MA) with 8.0 mm polycarbonate filter inserts were put in 24-well plates and coated with 40 μl Matrigel (BD Matrigel and serum-free DMEM were mixed in a ratio of 1:6) (BD Biosciences). 1-6 x 10^4^ cells suspended in serum-free DMEM medium were added to the upper compartment of the chamber. 700 μl CM were added to the lower compartment. After 48 h co-culture, the invaded cells were stained with 1% crystal violet and counted under a microscope.

### Quantitative PCR

Total RNA was extracted from cells using Trizol (Invitrogen). 2 μg RNA was used for reverse transcription by using TaKaRa kit (Dalian, China). For gene expression detection, Power SYBR Green Master Mix (ABI) was used and qPCR was performed on an ABI Prism 7900HT Sequence Detector System. The primers were designed by Primer Premier 5.0 and synthesized by BGI (Beijing, China). The sequences of the primers were listed in Table 1. The miRNAs Quantitation kit (GenePharma) was used to detect the expression of miR-101-3p, and U6 level was used for normalization.

**TABLE 1:** PCR primer sequences.

Primers		Sequence (5–3′)	Length of amplicons (bp)
TWIST1	Forward	GCC​AAT​CAG​CCA​CTG​AAA​GG	83
	Reverse	TGT​TCT​TAT​AGT​TCC​TCT​GAT​TGT​TAC​CA	
MMP9	Forward	TGT​ACC​GCT​ATG​GTT​ACA​CTC​G	97
	Reverse	GGC​AGG​GAC​AGT​TGC​TTC​T	
N-cadherin	Forward	AGC​CAA​CCT​TAA​CTG​AGG​AGT	136
	Reverse	GGC​AAG​TTG​ATT​GGA​GGG​ATG	
E-cadherin	Forward	CGA​GAG​CTA​CAC​GTT​CAC​GG	119
	Reverse	GGG​TGT​CGA​GGG​AAA​AAT​AGG	
Vimentin	Forward	AGT​CCA​CTG​AGT​ACC​GGA​GAC	97
	Reverse	CAT​TTC​ACG​CAT​CTG​GCG​TTC	
α-SMA	Forward	CACTGCCGCA TCCTCA TC	161
	Reverse	TGC​TGT​TGT​AGG​TGG​TTT​CA T	
VEGFA	Forward	AGG​GCA​GAA​TCA​TCA​CGA​AGT	75
	Reverse	AGG​GTC​TCG​ATT​GGA​TGG​CA	
GAPDH	Forward	TGC​ACC​ACC​AAC​TGC​TTA​GC	87
	Reverse	GGCA TGGACTGTGGTCA TGAG	
miR-101-3p	Forward	CAT​CGC​ACG​TAC​AGT​ACT​GTG​ATA	70
	Reverse	CTC​TGT​CTC​TCG​TCT​TGT​TGG​TAT	
U6	Forward	CGC​TTC​GGC​AGC​ACA​TAT​AC	87
	Reverse	TTC​ACG​AAT​TTG​CGT​GTC​ATC	

MMP9, matrix metallopeptidase 9; VEGFA, vascular endothelial growth factor A; α-SMA, α-smooth muscle actin; GAPDH, Glyceraldehyde-3-phosphate dehydrogenase.

### Western Blot Analysis

Western blottings were performed as previously described (Zhang Q. et al., 2020). Cells were treated with RIPA lysis buffer (Beyotime Biotechnology, China) containing protease inhibitor (Sigma- Aldrich) for 30 min on ice. Cell lysate was separated by 12% SDS-PAGE and transferred onto nitrocellulose membranes. The membranes were blocked with 5% milk in Tris-buffered saline with 0.5% Tween 20 (TBST), then were probed with primary antibodies and HRP-conjugated secondary antibodies. The blots were visualized with the ECL Western Blotting System (ThermoFisher Scientific).

### Transfection and Luciferase Assay

CAFs were transfected with miR-101-3p mimics, miR-NC, miR-101-3p inhibitor, inhibitor NC using lipofectamine 2000 (Invitrogen, Carlsbad, CA) following the manufacturer’s instruction. Briefly, the wild-type (wt) and mutant (mt) 3′-UTR of human VEGFA were cloned into pGL3 luciferase vector (Promega, Madison, WI). 293T cells were plated in 12-well plates and transfected with 100 nM miRNAs mimics or inhibitor, and 400 ng wt or mt VEGFA 3′-UTR plasmid, and 40 ng pRL-SV40 (Invitrogen) as an internal control. Luciferase activity were detected 24 h after transfection using Dual-luciferase Reporter Assay System (Promega).

### Enzyme-Linked Immunosorbent Assay

CAFs were transfected with miR-101-3p mimics, miR-NC, miR-101-3p-inhibitor or inhibitor NC for 24 h. The supernatants were collected to detect VEGFA using an human VEGFA ELISA Kit (RayBiotech Life, GA) following the manufacturer’s instructions.

### Tumor Xenograft Experiment

To establish stable cell lines, CAFs were transduced with lentiviruses expressing pre-miR-101-3p or negative control (CAFs/miR-101-3p, CAFs/miR-NC), and selected by puromycin. Female BALB/c nude mice (5-week-old) were obtained from the Cancer Institute of the Chinese Academy of Medical Science (Beijing, China). Mice were maintained under specific pathogen-free conditions, and randomly divided into 3 groups including 3 × 10^6^ A549-luc (expressing luciferase) alone, 3 × 10^6^ A549-luc cells and 6 × 10^6^ CAF-miR-NC (ratio 1:2) cells, or 3 × 10^6^ A549-luc and 6 × 10^6^ CAF-miR-101-3p cells (ratio 1:2). Cells were subcutaneously injected into the flank of mice. The experiment was conducted for 5 weeks. Tumor growth was measured by IVIS Imaging System (Xenogen Corporation, Alameda, CA). The animal experiments were conducted in accordance with the Tianjin Medical University Institutional Animal Care and Use Committee guidelines.

### Statistical Analysis

Data are presented as the means ± SEM. The differences between two groups were analyzed by student’s *t*-test, and the differences among more than two groups were analyzed by one-way analysis of variance. Analysis was performed using SPSS21.0. The *P* value less than 0.05 was considered statistically significant.

## Results

### CAFs Enhanced Migration and Invasion of NSCLC Cells

**FIGURE 1:**
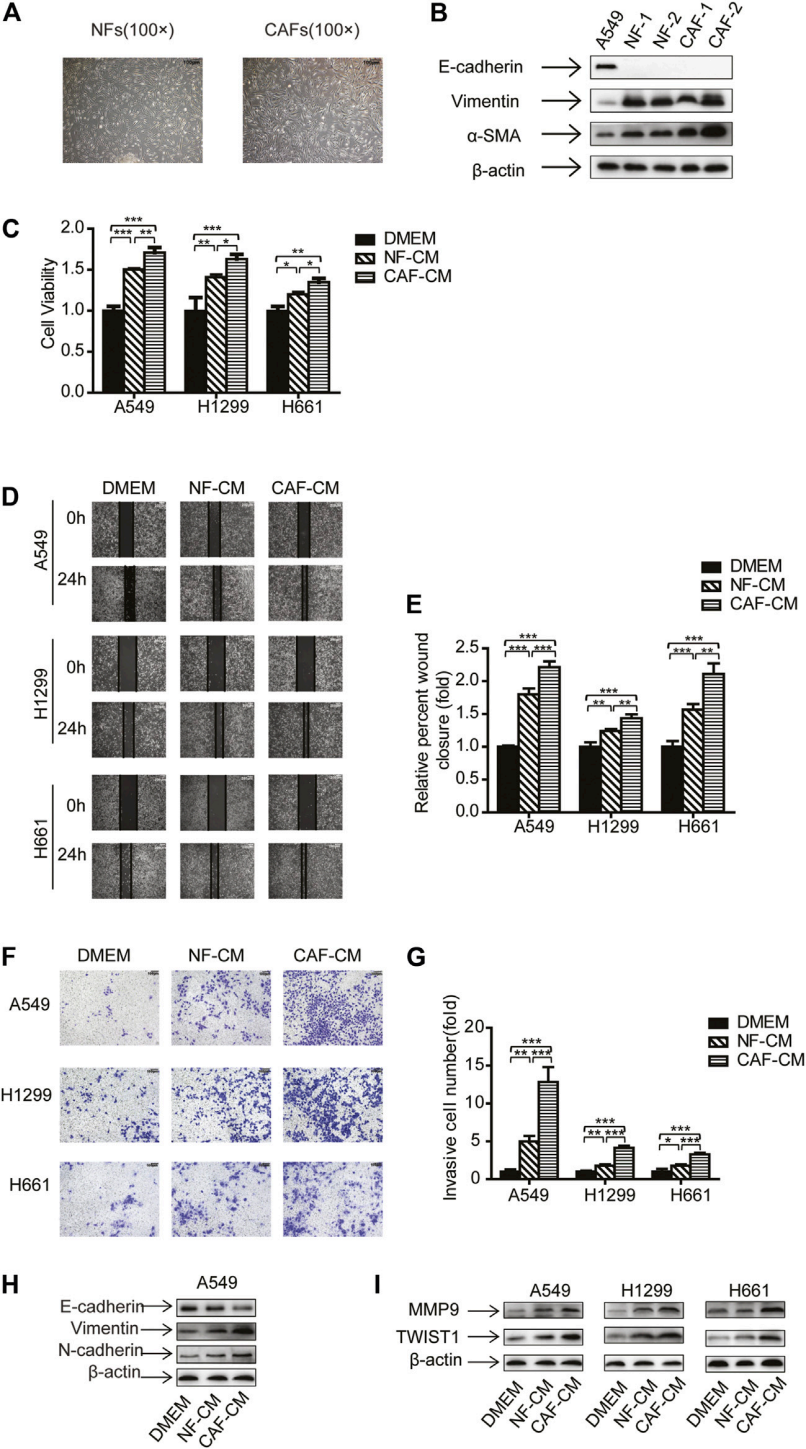
CAFs enhanced migration and invasion of NSCLC cells. **(A)** The morphology of cultured primary NFs and CAFs. Scale bar, 100 μM **(B)** The gene expression was detected by Western blots. **(C)** Lung cancer cells were cultured in NF-CM or CAF-CM. Cell growth was examined by CCK-8 kit after 48 h. **(D,E)** Cell invasion was detected by wound healing assay after 24 h. Scale bar, 200 μM **(F,G)** Cell migration was examined by transwell assay after 48 h. Scale bar, 100 μM **(H,I)** The gene expressions were detected by Western blots after 48 h. Values represent the mean ± SD from 3 independent experiments. There were three replicates in each independent experiment. *p < 0.05; **p < 0.01; ***p < 0.001.

In order to investigate CAFs’ effect on NSCLC cells, we have performed experiments on NSCLC cell lines A549, H1299 and H661, which represent adenocarcinoma, carcinoma and large cell carcinoma, respectively. CAFs and NFs were isolated and characterized as our previous work (Wang et al., 2017). Both CAFs and NFs displayed spindle-like shape, and morphology showed no notable difference between CAFs and NFs (Figure 1A). We then detected the fibroblast markers by Western blotting analysis. Different from A549 cells which are epithelial cells, CAFs and NFs did not express E-cadherin, but expressed vimentin and α-SMA. Notably, CAFs possessed higher level of α-SMA than NFs (Figure 1B).

Next, NF-CM and CAF-CM were collected and cultured lung cancer cells, and cell proliferation was examined by CCK-8 kit. Figure 1C showed that both CAFs and NFs promoted lung cancer cell growth, and CAFs was more effective than NFs. To assess the effect of CAFs on migration and invasion of NSCLC cells, NSCLC cells were treated with NF-CM and CAF-CM, DMEM medium was used as a control, then wound healing assay and transwell assay was performed. The results of wound healing assay showed that the adhesive rates for A549, H1299 and H661 cells were increased to 1.80-, 1.24- and 1.56-fold of control by NFs, respectively. When cultured with CAF-CM, the migration levels were increased to 2.21-, 1.43- and 2.11-fold of control, respectively (Figures 1D,E). Consistent with the migration assay, the transwell assay indicated that both NFs and CAFs stimulated cell invasion, and CAFs were more effective (Figures 1F,G).

EMT is a key stage in tumor metastasis process (Welch and Hurst, 2019), we further investigated the EMT process when lung cancer cells were treated with NF-CM or CAF-CM. The expression of epithelial marker E-cadherin decreased whereas the expression of mesenchymal marker vimentin and N-cadherin increased (Figure 1H). Given that metastasis is mediated by metastasis-related genes, we then examined the effect of CAFs on metastasis-related genes MMP9 and Twist1. Figure 1I showed that NF-CM and CAF-CM upregulated the expression of MMP9 and Twist1, and CAFs had stronger effect. Taken together, our data suggested that CAFs enhanced the migration and invasion of NSCLC cells through regulating EMT process and metastasis-related genes.

### MiR-101-3p was Downregulated in CAFs and Suppressed CAFs Activation

**FIGURE 2:**
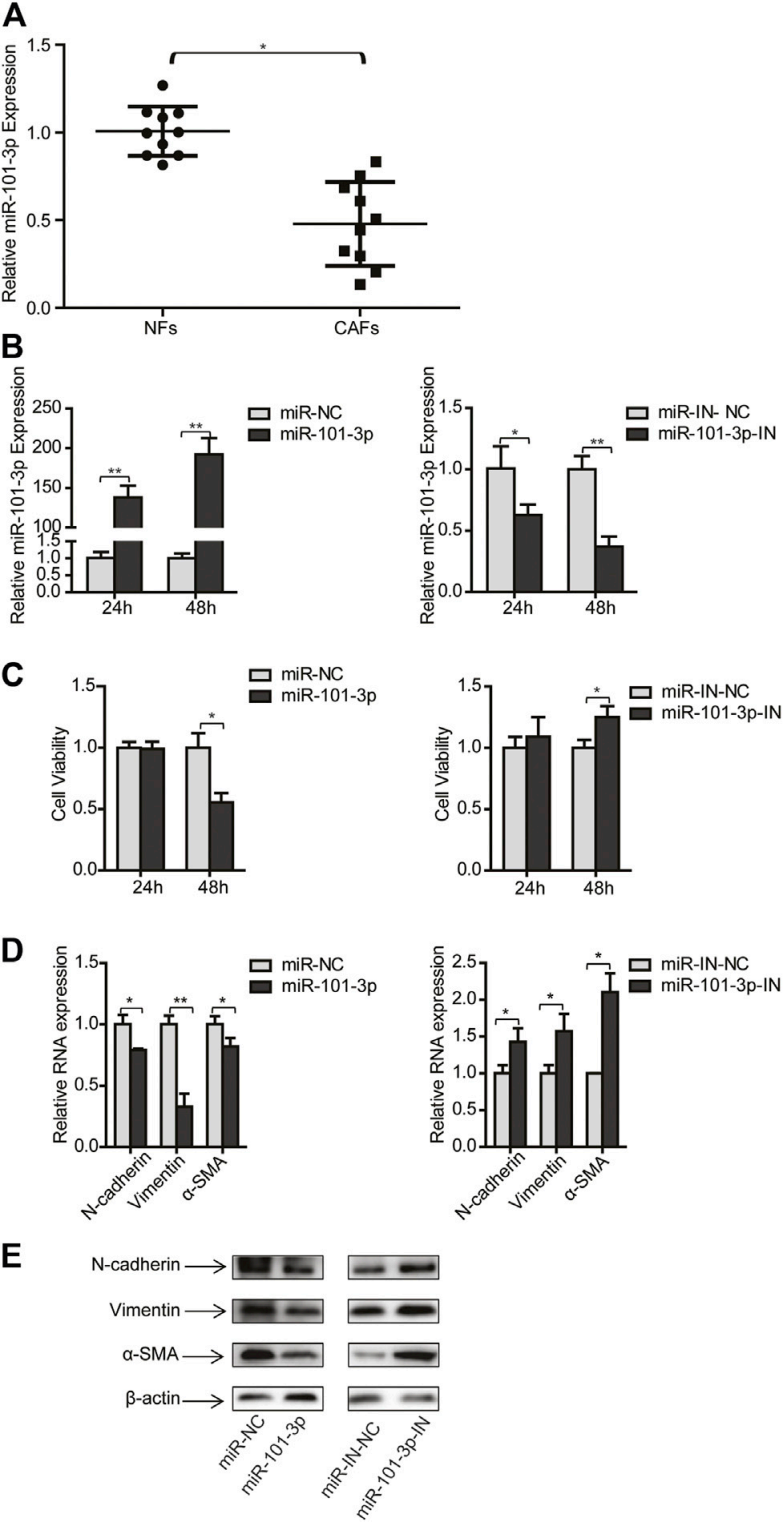
MiR-101-3p suppressed CAFs activation. **(A)** MiR-101-3p expression in NFs and CAFs was detected by qPCR (*n* = 10). CAFs were transfected with 100 nM of miR-101-3p mimics or inhibitors. **(B)** MiR-101-3p expression in CAFs was detected by qPCR. **(C)** Cell viability was examined by CCK-8 kit. **(D)** Gene expression was detected by qPCR. **(E)** Gene expression was detected by Western blots. Values represent the mean ± SD from 3 independent experiments. *p < 0.05; **p < 0.01; ***p < 0.001.

Numerous studies have shown that miR-101-3p is dysregulated in various types of tumors and suppress tumor growth including NSCLC (Li Z. et al., 2020; Wang et al., 2020; Zeng et al., 2020). However, the role of miR-101-3p in CAFs is unclear. We first evaluated the miR-101-3p level in CAFs, and found that miR-101-3p was down-regulated in CAFs compared with NFs in NSCLC (Figure 2A). We then manipulated the miR-101-3p expression in CAFs by transfecting miR-101-3p mimics or inhibitor, and examined its effect on CAFs proliferation. MiR-101-3p had no remarkable effect after 24 h transfection, however, after 48 h transfection, miR-101-3p mimics suppressed CAFs growth whereas miR-101-3p inhibitor promoted CAFs growth (Figures 2B,C).

To determine whether miR-101-3p play a role in CAFs activation, we overexpressed or knocked down miR-101-3p, then detected the expression of CAFs markers including N-cadherin, vimentin and α-SMA. At transcription level, miR-101-3p repressed mRNA expression of N-cadherin, vimentin and α-SMA expression, respectively. On the contrary, miR-101-3p inhibition enhanced mRNA expression of these genes. In line with this finding, Western blotting results showed that miR-101-3p also affected CAFs markers expression at protein level (Figures 2D,E). Collectively, these data demonstrated that miR-101-3p repressed CAFs activation.

### MiR-101-3p Mediated the Effect of CAFs on Migration and Invasion of NSCLC Cells

**FIGURE 3:**
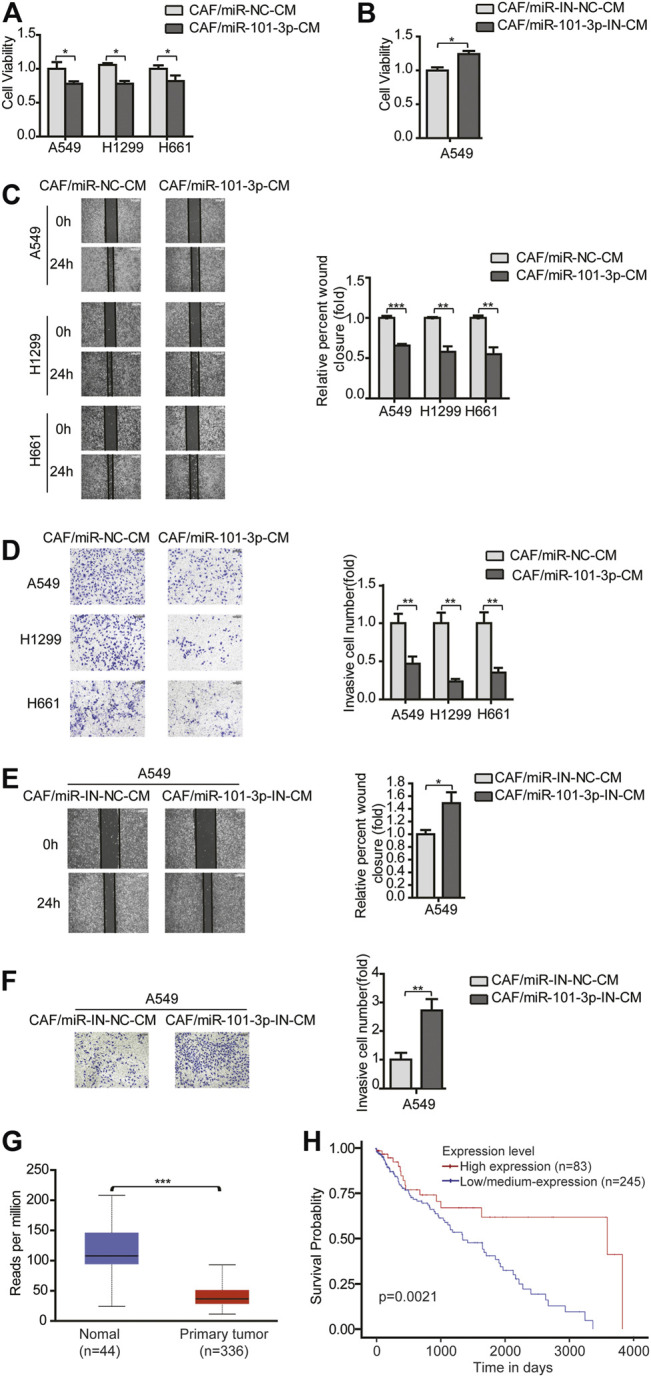
MiR-101-3p mediated the effect of CAFs on migration and invasion of NSCLC cells **(A,B)** CAFs were transfected with 100 nM of miR-101-3p mimics or inhibitor. CM was collected and cultured lung cancer cells. Lung cancer cell viability was examined by CCK-8 kit. **(C,D)** CAFs were transfected with miR-101-3p mimics. CM was collected and culture lung cancer cells. Cell invasion was detected by wound healing assay, and cell migration was examined by transwell assay. **(E,F)** CAFs were transfected with miR-101-3p inhibitor. Cell invasion was detected by wound healing assay, and cell migration was examined by transwell assay. **(G)** MiR-101-3p expression in non-tumor specimens (*n* = 44) and NSCLC specimens (*n* = 336) based on TCGA database. **(H)** OS rate in NSCLC patients divided into high and low/medium expression of miR-101-3p (*n* = 328) based on TCGA database. Values represent the mean ± SD from 3 independent experiments. *p < 0.05; **p < 0.01; ***p < 0.001.

Given that miR-101-3p repressed CAFs activation, this provided rationale for further evaluating the role of miR-101-3p in CAFs’ effect on NSCLC cells. CAFs were transfected with miR-101-3p mimics or inhibitors, and CM was collected and cultured lung cancer cells. Figures 3A,B showed that miR-101-3p inhibited lung cancer cell proliferation whereas miR-101-3p down-regulation stimulated lung cancer cell proliferation. Furthermore, we verified the role of miR-101-3p in lung cancer cell invasion. When miR-101-3p was up-regulated in CAFs, the promoting effects of CAFs on lung cancer cell migration and invasion were mitigated significantly, on the contrary, when miR-101-3p was down-regulated in CAFs, the promoting effect of CAFs on lung cancer cell migration and invasion were enhanced (Figures 3C–F). Our results demonstrated that miR-101-3p mediated the effect of CAFs on migration and invasion of NSCLC cells.

Furthermore, we investigated the clinical implication of miR-101-3p level in NSCLC patients by using TCGA database. A cohort of 336 NSCLC specimens and 44 non-tumor specimens were analyzed. MiR-101-3p was significantly down-regulated in lung cancer tissues compared with non-cancerous tissues (Figure 3G). For Kaplan–Meier analysis, we found that low/medium-expression of miR-101-3p was associated with poor OS rate (Figure 3H, *p* = 0.0021). These findings suggested that miR-101-3p might serve as a prognostic marker for OS in NSCLC patients.

### MiR-101-3p in CAFs was Responsible for the Regulation of EMT and AKT/eNOS Signaling Pathway in Lung Cancer Cells

**FIGURE 4:**
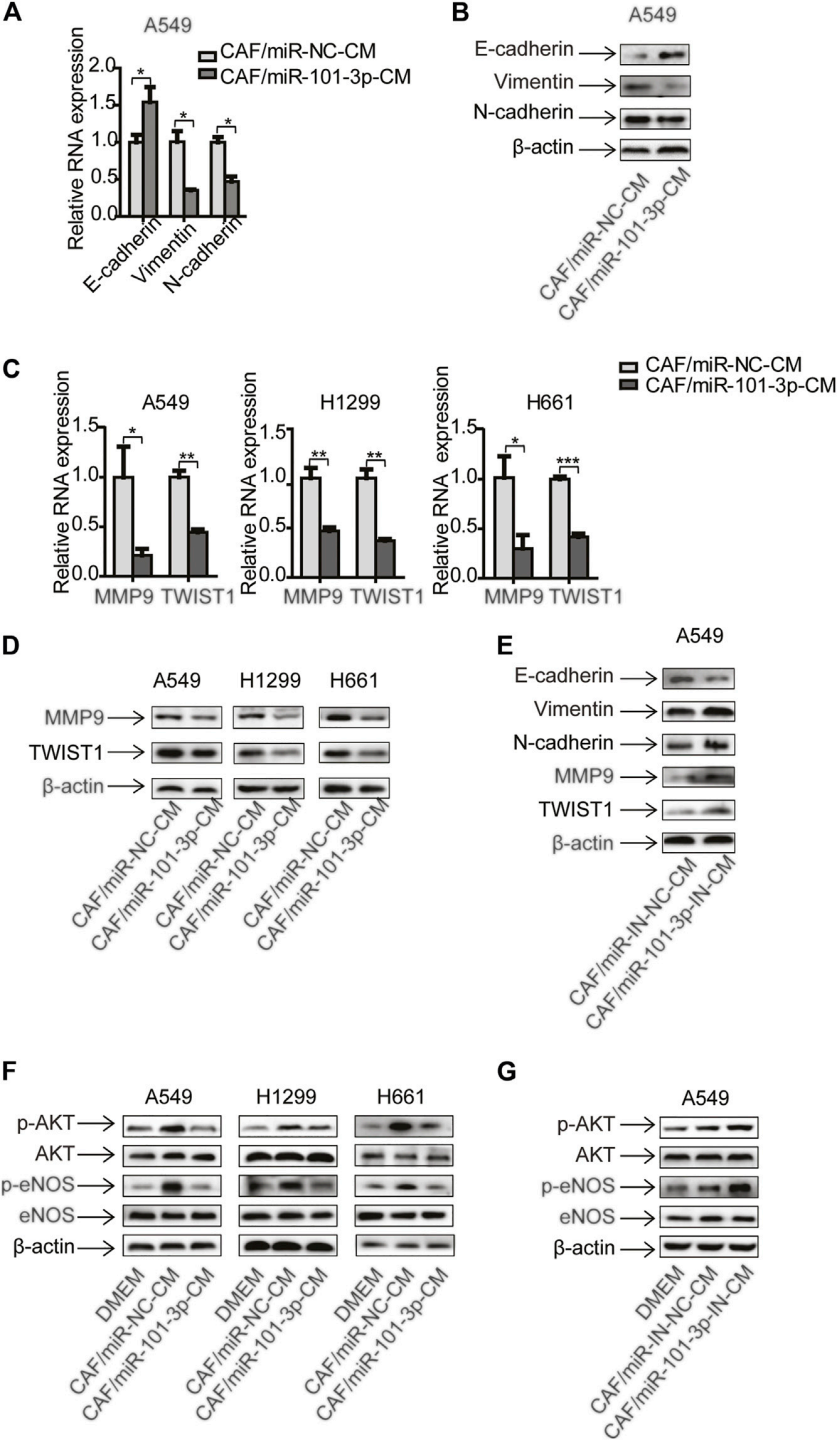
MiR-101-3p in CAFs was responsible for the regulation of EMT and AKT/eNOS signaling pathway in lung cancer cells CAFs were transfected with 100 nM of miR-101-3p mimics. CM was collected and cultured lung cancer cells. The expression of EMT marker genes in lung cancer cells was detected by qPCR **(A)** and Western blots **(B)**. The expression of metastasis-related genes in lung cancer cells was detected by qPCR **(C)** and Western blots **(D)**. CAFs were transfected with 100 nM of miR-101-3p inhibitor. CM was collected and culture lung cancer cells. The expression of genes in lung cancer cells was detected by Western blots **(E)**. CAFs were transfected with miR-101-3p mimics or inhibitor. CM was collected and culture lung cancer cells. The expression of genes in lung cancer cells was detected by Western blots after 48 h **(F,G)**. Values represent the mean ± SD from 3 independent experiments. *p < 0.05; **p < 0.01; ***p < 0.001.

Since miR-101-3p played a role in CAFs’ effect on cell invasion, we further explored the underling mechanism. Given that CAFs regulated EMT process and metastasis-related genes in NSCLC cells (Figures 1H,I), we therefore investigated whether miR-101-3p was involved. MiR-101-3p was overexpressed in CAFs and conditioned medium was collected and treated lung cancer cells. qPCR and Western blot analysis showed that E-cadherin was up-regulated whereas vimentin and N-cadherin were down-regulated (Figures 4A,B). Figures 4C,D indicated that metastasis-related genes MMP9 and Twist1 were decreased at both mRNA and protein level. Moreover, we repressed miR-101-3p expression in CAFs by using miR-101-3p inhibitor, as expected, E-cadherin was down-regulated whereas vimentin and N-cadherin were up-regulated, and metastasis-related genes were stimulated (Figure 4E). These results demonstrated that miR-101-3p attenuated CAFs’ effect on EMT process and metastasis-related genes activation in lung cancer cells.

Cumulative studies have reported that AKT signaling pathway plays an important role in tumor metastasis (Wan et al., 2013). We next examined the activation of AKT signaling pathway in this setting. Compared with DMEM, CAF-CM elevated the phosphorylation of AKT and eNOS in lung cancer cells, meanwhile, the total level of AKT and eNOS remained unchanged. However, miR-101-3p diminished CAFs’ effect (Figure 4F). Interestingly, when miR-101-3p was repressed in CAFs, the CAFs’ effect on AKT/eNOS pathway activation was boosted (Figure 4G). Taken together, our data illustrated that miR-101-3p down-regulation in CAFs is responsible for facilitating EMT program and AKT/eNOS pathway activation in lung cancer cells.

### VEGFA was a Direct Target of miR-101-3p

MiRNA executes its function *via* modulating its target genes. To search for miR-101-3p targets, we applied bioinformatics algorithm including miRTarBase and TargetScan. Among many predicted targets, VEGFA drew our attention because VEGFA is reported to be involved in tumor progression (Matsumoto and Ema, 2014), we therefore selected VEGFA as a candidate target for further investigation.

**FIGURE 5:**
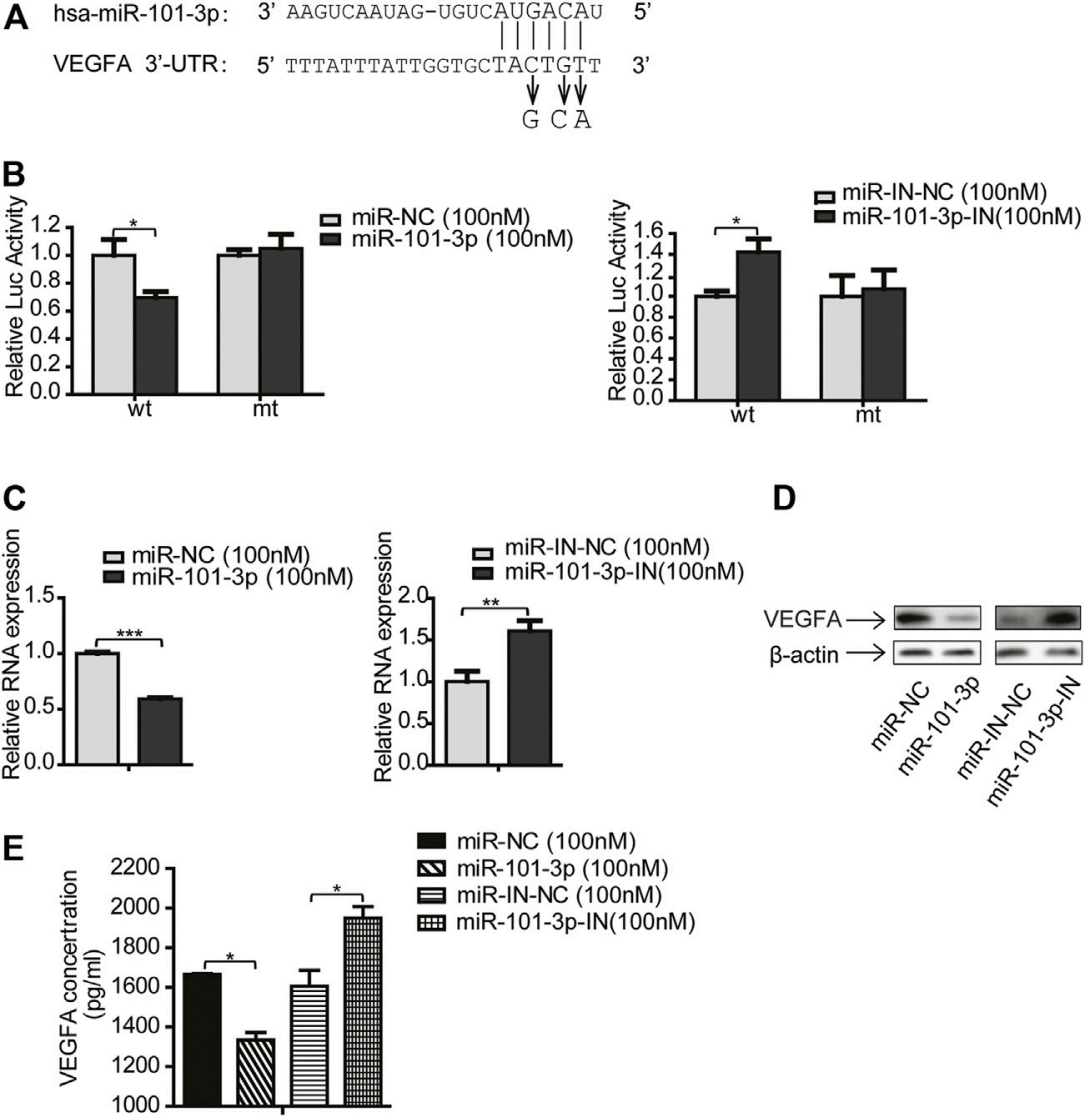
VEGFA was a direct target of miR-101-3p. **(A)** Putative miR-101-3p binding sites in 3′-UTR of VEGFA. Nucleotide changes for binding site mutation were indicated. **(B)** The reporter vectors containing the wild-type (wt) and mutant (mt) VEGFA 3′-UTR regions were co-transfected into 293T cells with miR-101-3p, miR-101-3p inhibitor and pRL-SV40 plasmid. The relative luciferase/pRL-SV40 activities were measured 24 h after transfection. **(C,D)** CAFs were transfected with miR-101-3p or miR-101-3p inhibitor. The mRNA and protein expression of VEGFA were detected by qPCR and Western blots 24 h after transfection. **(E)** The secreted VEGFA were detected by ELISA. Values represent the mean ± SD from 3 independent experiments. *p < 0.05; **p < 0.01; ***p < 0.001.

To determine whether VEGFA is a direct target of miR-101-3p, we performed dual-luciferase assay. We first cloned the putative target sequence (wild type, wt) of VEGFA 3′UTR and mutant sequence (mt) into pGL3 luciferase vector (Figure 5A), then transfected these plasmids along with miR-101-3p mimics into 293T cells. The luciferase assay results showed that miR-101-3p mimics significantly reduced the luciferase activities of wt 3′UTR vector, but had no effect on mt 3′UTR vector. In contrast, miR-101-3p inhibitor increased luciferase activities of wt 3′UTR vector (Figure 5B).

To further validate VEGFA as a target of miR-101-3p, we investigated whether VEGFA expression was regulated by miR-101-3p. CAFs were transiently transfected with miR-101-3p mimics or inhibitor, and VEGFA expression was detected by qPCR and Western blot. Figures 5C,D indicated that miR-101-3p suppressed VEGFA expression at both mRNA and protein levels, and miR-101-3p inhibition enhanced VEGFA expression. Since VEGFA was a cytokine, we further examined whether CAFs secreted VEGFA or not by ELISA assay. Our data revealed that CAFs secreted VEGFA into CAF-CM, and miR-101-3p effectively reduced VEGFA secretion by CAFs (Figure 5E). Collectively, these results demonstrated that VEGFA was a direct target of miR-101-3p.

### VEGFA Mediated the Effect of miR-101-3p on Migration and Invasion of Lung Cancer Cells

Since CAFs promote cancer progression *via* secreting cytokines and growth factors (Kalluri, 2016), and our study showed that CAFs secreted VEGFA (Figure 5E), we explored whether VEGFA play a role in CAFs mediated metastasis. We manipulated VEGFA level in CAF-CM by adding 20 ng/ml of human recombinant VEGFA or 5 μg/ml of VEGFA neutralizing antibody to cell cultures, and determined these effects on cell growth, migration and invasion.

**FIGURE 6:**
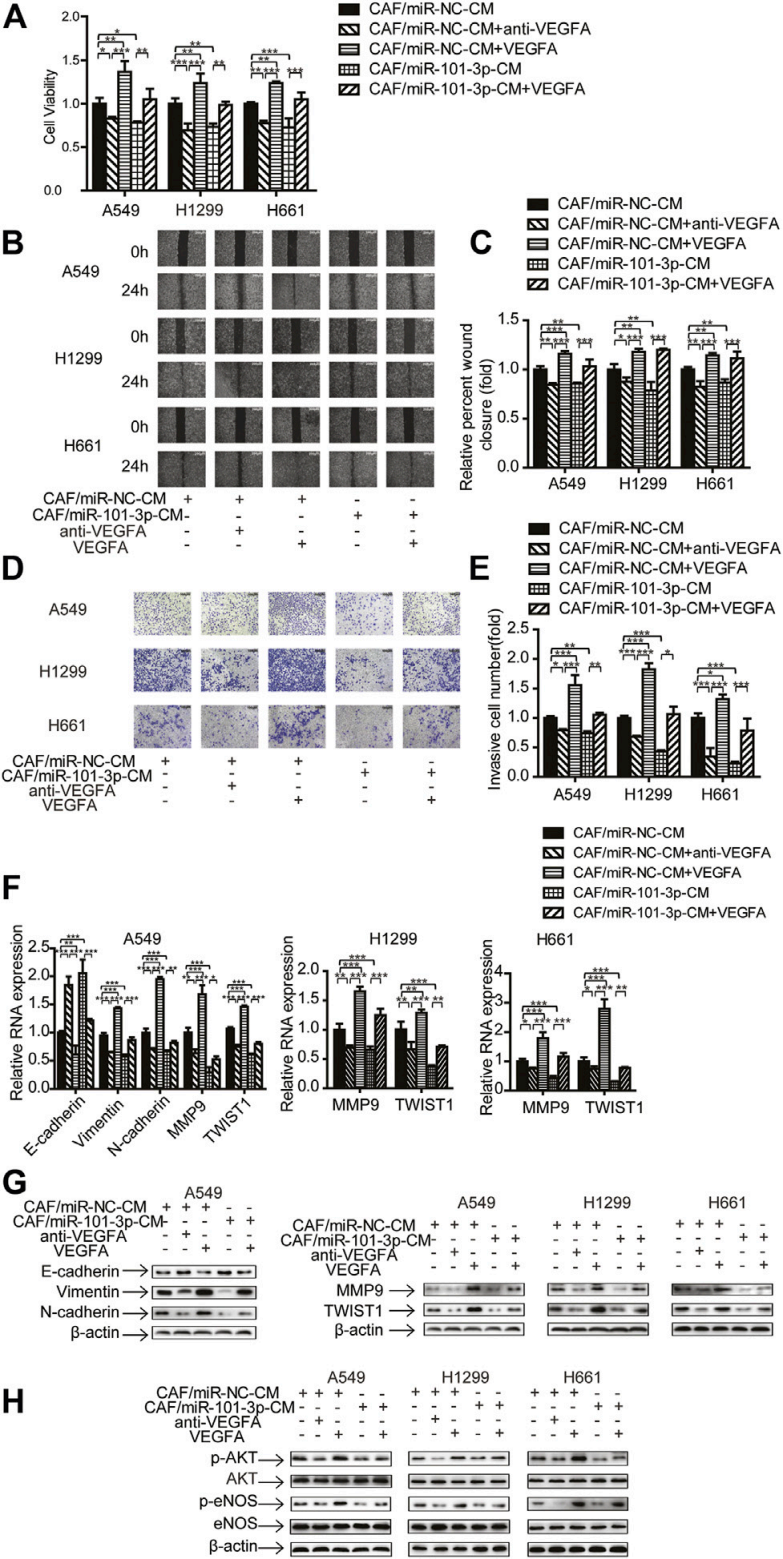
VEGFA mediated the effect of miR-101-3p on migration and invasion of lung cancer cells Lung cancer cells were cultured in CAF/miR-NC-CM or CAF/miR-101-3p-CM. 20 ng/ml of human recombinant VEGFA or 5 μg/ml of VEGFA neutralizing antibody were added. **(A)** Cell viability was examined by CCK-8 kit. **(B,C)** Cell migration was detected by wound healing assay. **(D,E)** Cell invasion was detected by transwell assay. **(F)** Gene expression was detected by qPCR. **(G,H)** Gene expression was detected by Western blots. Values represent the mean ± SD from 3 independent experiments. *p < 0.05; **p < 0.01; ***p < 0.001.

Figure 6A showed that recombinant VEGFA boosted CAFs’ effect on cell proliferation, whereas VEGFA neutralizing antibody mitigated CAFs’ effect. Interestingly, when recombinant VEGFA was added to CAF-miR-101-3p-CM, the inhibitory effect of miR-101-3p on cell growth was reversed. We next examined the role of VEGFA in lung cancer cell migration and invasion. Both wound healing assay and transwell assay revealed that VEGFA enhanced CAFs’ effect on cell migration and invasion, and VEGFA reversed the inhibition of miR-101-3p on cell migration and invasion (Figures 6B–E). Moreover, the roles of VEGFA in EMT process and metastasis-related genes of lung cancer cells were also investigated. As expected, VEGFA promoted the enhancement of CAFs on EMT process and metastasis-related genes, and VEGFA attenuated miR-101-3p′s inhibitory effect (Figures 6F,G). Furthermore, Figure 6H showed that VEGFA also affected miR-101-3p′ effect on AKT/eNOS signaling pathway in lung cancer cells. Taken together, these results demonstrated that CAFs promoted metastasis potential of lung cancer cell *via* miR-101-3p regulated VEGFA secretion.

### CAFs Promoted Tumor Growth *via* Downregulation of miR-101-3p *in vivo*

**FIGURE 7:**
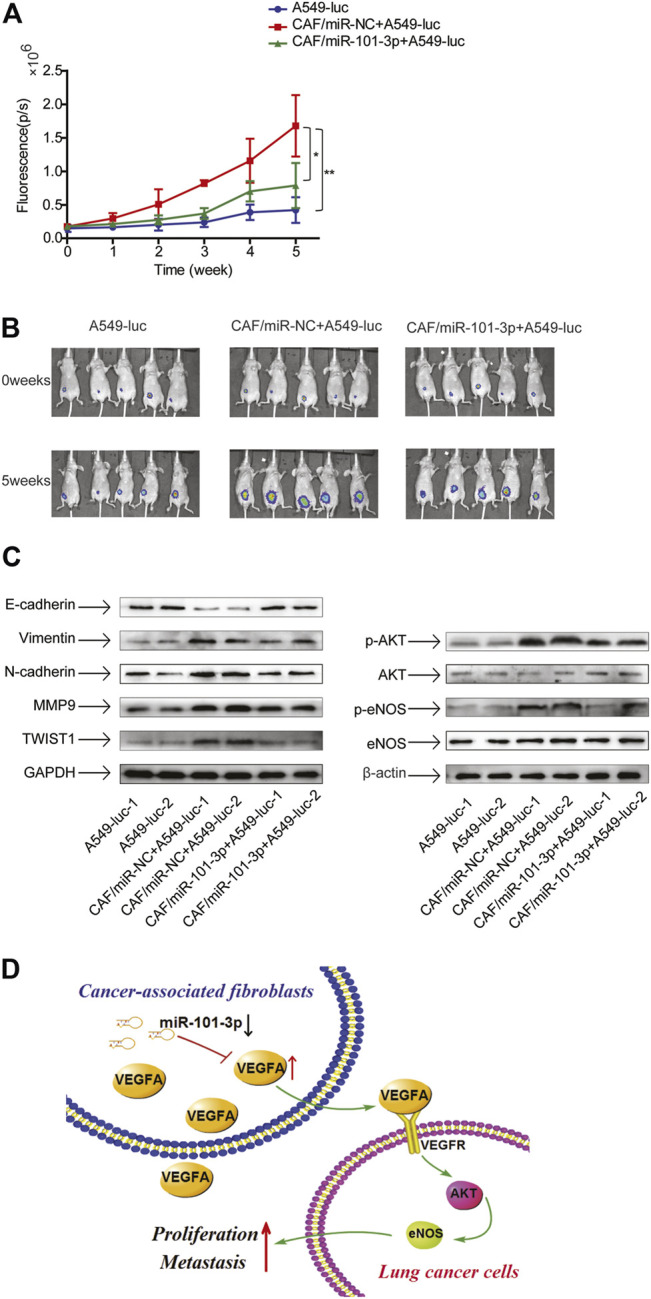
CAFs promoted tumor growth *via* downregulation of miR-101-3p *in vivo* 3 × 10^6^ A549-luc cells alone, or 3 × 10^6^ A549-luc cells and 6 × 10^6^ CAF-miR-NC cells, or 3 × 10^6^ A549-luc and 6 × 10^6^ CAF-miR-101-3p cells were subcutaneously injected into the flank of mice. **(A,B)** Tumor growth was measured by IVIS Imaging System at indicated time points. **(C)** Protein expressions were detected by Western blots. **(D)** Scheme illustrating the cross-talk between CAFs and lung cancer cells mediated by miR-101-3p/VEGFA/Akt axis. *p < 0.05; **p < 0.01.

Since our study showed that CAFs stimulated lung cancer cell growth *in vitro*, we next evaluated their effect on tumor growth *in vivo*. Mice were randomly divided into 3 groups consisting of A549-Luc cells alone, A549-Luc cells combined with CAFs-miR-NC, and A549-Luc cells combined with CAFs-miR-101-3p. Cells were subcutaneously injected into the flank of mice. Tumor growth was observed in successive 5 weeks by IVIS Imaging System. CAFs stimulated tumor growth significantly, the average size of tumors in A549-Luc cells combined with CAFs-miR-NC group was 3.77-fold of A549-Luc cells alone group. Notably, the average size of tumors in A549-Luc cells combined with CAFs-miR-101-3p group was reduced to 1.87-fold of A549-Luc cells alone group, indicating that miR-101-3p mitigated CAFs’ effect (Figures 7A,B).

Next, we investigated the EMT status and AKT pathway in tumors. The tumor tissues were dissected and related molecules were examined by Western blottings. In line with our findings of *in vitro* study, CAFs promoted EMT progress, upregulated metastasis-related genes, and activated AKT pathway, meanwhile, miR-101-3p attenuated these effects (Figure 7C). Collectively, our results demonstrated that CAFs facilitated tumor growth through promoting EMT and AKT pathway by downregulation of miR-101-3p.

## Discussion

Tumor progression depends on the communication between TME and tumor cells. CAFs are major components of tumor stroma, hence in the present study, we established the culture of CAFs derived from human lung cancer tissues, and investigated their effects on metastasis potential of lung cancer cells. We found that CAFs enhanced migration and invasion of lung cancer cells. Further study showed that CAFs induced EMT and stimulated metastasis-related genes in lung cancer cells.

Despite many studies are focused on the tumor promoting effect of CAFs-derived cytokines, the upstream regulators of cytokine release in CAFs is largely unknown. MiRNAs regulate the expression of target genes; however, very few studies showed the role of miRNAs in regulating growth factors release from CAFs. Zhang S. et al. (2020) reported that in human placental site trophoblastic tumor (PSTT), miR-363 negatively regulated EGR1 in CAFs, leading to the reduction of Angiopoietin-1 secretion which contributed to the angiogenesis of PSTT. Another study showed that miR-101 inhibited the interaction between CAFs and cancer cells *via* abrogating CXCL12 release from CAFs (Zhang et al., 2015). We are also interested in miRNA’s role in regulation of growth factor secretion from CAFs. Zhang et al. (2015) compared the miRNA expression profile between CAFs and NFs in lung cancer. Among differentially expressed miRNAs, downregulated miR-101-3p drew our attention, since several studies revealed that miR-101-3p is involved in metastasis. MiR-101-3p induced M1 to M2 macrophage-type conversion and enhanced cell migration of breast and ovarian (Zhao et al., 2021). MiR-101-3p promoted hepatocellular carcinoma carcinogenesis by targeting glycogen phosphorylase B (Cui et al., 2020). Li et al. (2019) reported that miR-101-3p targeted TRIM44 to inhibit EMT and suppress glioblastoma cell proliferation metastasis. While these functional studies of miR-101-3p were focused on the role of miR-101-3p in cancer cells, we investigated the role of miR-101-3p in CAFs. Our results showed that miR-101-3p was downregulated in CAFs compared to NFs. Ectopic overexpression of miR-101-3p suppressed CAFs activation, and abrogated the promoting effect of CAFs on migration and invasion of NSCLC cells.

MiRNAs exert their function by negatively regulating their target genes. There are several miR-101-3p target genes have been reported, which are involved in different biological processes. MiR-101-3p promotes apoptosis of oral cancer cells by targeting BICC1 (Wang et al., 2020), induces dysfunction of vascular endothelial cell by targeting tet methylcytosine dioxygenase 2 (Chen et al., 2020), suppresses proliferation and metastasis of glioblastoma cells *via* targeting TRIM44 (Li et al., 2019). In the present study, for the first time, we identified VEGFA as a novel target of miR-101-3p. Angiogenesis has essential roles in tumor invasion and progression. VEGFA is one of the key regulators of angiogenesis, it is responsible for many angiogenesis-related diseases including cancer (Matsumoto and Ema, 2014), it also mediates the effect of miRNAs in cancer. MiR-205-5p is downregulated in gastric cancer tissues, it suppresses proliferation and angiogenesis in gastric cancer by reducing the expression of VEGFA and FGF1 (Zhang J. et al., 2021). MiR-125a-5p inhibits colorectal cancer growth and invasion, VEGFA is a direct target of miR-125a-5p and could reverse the inhibitory effect of miR-125a-5p (Yang et al., 2018). Qu et al. (2017) reported that miR-16-5p overexpression attenuates tumor growth and reduced VEGFA expressions in breast cancer cells. Here we demonstrated that miR-101-3p inhibited VEGFA secretion from CAFs, and VEGFA mediated the effect of CAFs on migration and invasion of lung cancer cells.

EMT is a critical step for tumor metastasis. During EMT process the loss of epithelial phenotypes and gain of mesenchymal phenotype facilitate invasion and metastasis of cancer cells. Several studies also indicate that CAFs promote EMT transition in cancer cells. CAFs induce EMT and inhibits apoptosis to promote Hepatocellular Carcinoma Progression (Wang C. et al., 2021). Qin et al. (2021) reported that CAFs induces EMT and enhances gastric cancer migration and migration *via* the CXCL12-CXCR4 axis. In lung cancer, CAFs-derived SDF-1 induces EMT *via* CXCR4/β-catenin/PPARδ signaling (Wang Y. et al., 2021). Our results revealed that miR-101-3p is responsible for CAFs’ promoting effect on EMT process in lung cancer cells.

Metastasis-related genes play important role in tumor metastasis; we thus examined the effect of CAFs on metastasis-related genes MMP9 and twist1 in lung cancer cells. MMP9 is a member of matrix metalloproteinase (MMP) family, which degrades the components of extracellular matrix to promote cancer cell invasion and metastasis. CAFs can increase MMP9 expression and enhance growth and metastasis of melanoma. This pro-tumoral activity is dependent on CD38 activation in CAFs (Ben Baruch et al., 2020). Zhang Y. et al. (2020) reported that estrogen stimulates CAFs to secret IL-6, which regulates MMP9 expression, and promotes gastric cancer cell proliferation and invasion. Teng et al. (2016) study showed that CAFs promoted migration and invasion of endometrial cancer cells through SDF-1α/CXCR4 mediated MMP9 secretion. Twist1 is an EMT-regulating transcription factor, it plays key role in metastasis. Only limited studies showed that CAFs modulated Twist1 in cancer cells. CAFs released CXCL12 activates AKT and regulates Twist1 to promote EMT in esophageal cancer cells (Zhu et al., 2019). In MET-unamplified gastric cancer cells, CAFs-derived HGF activates HGF/STAT3/Twist1 pathway to enhance cell migration and invasion (Ding et al., 2018). In this study, we found that downregulation of miR-101-3p in CAFs upregulated the expression of MMP9 and Twist1 in lung cancer cells.

We further explore the signaling pathway involved in CAFs’ promoting effect on invasion of lung cancer cells. Nitric Oxide (NO) regulates many biological functions. NO is produced by three different NO synthase (NOS) enzymes, including neuronal NOS (nNOS), inducible NOS (iNOS) and eNOS (Shu et al., 2015). Recent studies indicate that AKT/eNOS pathway is responsible for tumorigenesis and cancer development. Glucose-regulated protein 94 (GRP94) is highly expressed in hepatocellular carcinoma (HCC), it promotes HCC progression through modulating AKT pathway and eNOS levels (Huang et al., 2016). The suppressor of MEK null (sMEK1) protein is an anti-angiogenic factor. sMEK1 inhibits ovarian cancer cell growth and migration *via* suppressing VEGFR-2-mediated Akt/eNOS/HIF-1α pathway (Kim et al., 2015). Chatterjee et al. (2021) reported that PARP inhibitor Veliparib enhanced curcumin’s inhibitory effect on oral cancer. Mechanistic study showed that curcumin-Veliparib combination suppresses angiogenesis through attenuating PI3K/AKT/eNOS signaling pathway. Consistent with these studies, our results demonstrated that downregulation of miR-101-3p in CAFs also activated AKT/eNOS signaling pathway in lung cancer cells.

In summary, our results demonstrated that miR-101-3p was downregulated in lung cancer-associated CAFs. Further mechanistic study showed that downregulation of miR-101-3p in CAFs increased VEGFA secretion, facilitating the metastasis potential of lung cancer cells *via* activation of Akt/eNOS signaling pathway (Figure 7D). Our finding suggests that modulation of miR-101-3p in CAFs might be a promising strategy for lung cancer treatment.

## Data Availability

The original contributions presented in the study are included in the article/Supplementary Material, further inquiries can be directed to the corresponding authors.
